# The Use of the S-MART Tourniquet in Hand Surgery: A Safe and Effective Way to Provide a Bloodless Field

**DOI:** 10.1155/2014/402184

**Published:** 2014-01-02

**Authors:** O. Templeton-Ward, J. Feher, P. Davey

**Affiliations:** Kingston Hospital NHS Trust, Galsworthy Road, Kingston upon Thames, Surrey KT2 7QB, UK

## Abstract

We have retrospectively reviewed our use of the S-MART sterile silicon ring self-exsanguinating tourniquet in 300 consecutive minor hand surgical procedures. A total of 3 postoperative complications were identified, only 1 of which was directly related to the tourniquet's use. We outline the reasons of why we feel that this device provides a safe and effective bloodless field and the benefits of its use.

## 1. Introduction

The S-MART tourniquet (OHK Medical Devices, Newark, NJ) is a novel single-use tourniquet, which provides exsanguination of the limb, arterial occlusion, and application of sterile stockinet in one device. Its use in extremity surgery has been documented, but the largest series to date has been 51 patients [1, 2].

**Figure 1:**
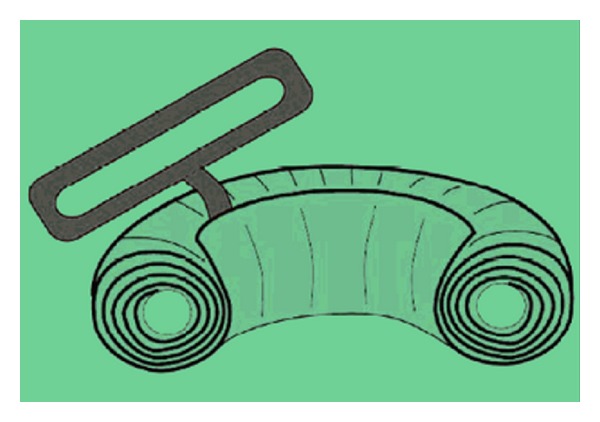
Cross section of the S-MART tourniquet.

**Figure 2:**
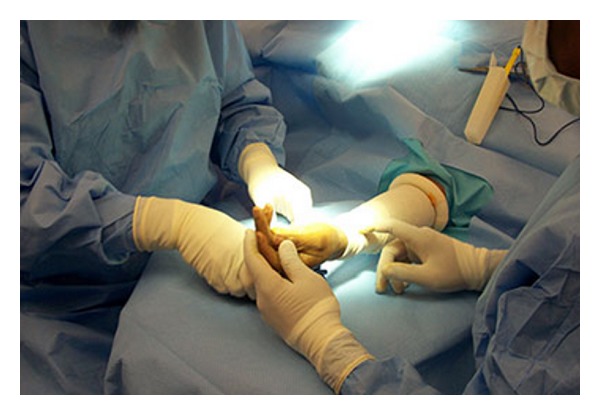
S-MART after application.

The S-MART tourniquet works by virtue of a core silicon ring which provides the pressure required to expel luminal blood during application and maintain arterial occlusion throughout the procedure once in situ. This ring is wrapped in sterile stockinet with pull handles attached to facilitate application.  Figure 1 shows a cross section of the S-MART and Figure 2 shows its application.

In a recent article, Noordin et al. [3] criticized the use of nonpneumatic ring type tourniquets such as the S-MART in nonbattlefield settings claiming that their use may increase the occurrence of tourniquet-related adverse events. The article discussed two particular adverse events: tourniquet-related nerve injury and skin blistering.

We have been using the S-MART tourniquet for all appropriate hand and wrist procedures in our day surgery department for a number of years; anecdotally we have not noticed any increase in the complications suggested by Noordin et al. and thus felt it would be worthwhile to review a cohort of patients to ensure this was the case; we report our experience. 

## 2. Methods

Our cohort included the last 300 patients undergoing one of the 4 most common procedures in our department: carpal tunnel decompression, De Quervain's decompression, trigger finger release, and ganglion excision with a minimum of 6-month follow-up (prior to January 2010). Exclusion criteria were incomplete notes, no documented postoperative follow-up, inability to use S-MART tourniquet (limb circumference too great), and preexisting soft tissue damage or neurological lesion (other than carpal tunnel syndrome) affecting the limb concerned. All procedures were carried out under the supervision of the senior author (P. Davey); 50 patients were excluded (47 missing or incomplete notes, 1 no follow-up, 0 pneumatic tourniquet used, and 1 preexisting soft tissue or nerve injury). We retrospectively reviewed all medical notes relating to our cohort and documented the procedure type, tourniquet time, adequacy of the bloodless operative field, and any intraoperative or postoperative complications, with particular reference to neuropraxia or skin damage at the tourniquet site.

## 3. Results

250 patients fulfilled the inclusion criteria and were reviewed for the purpose of this study, 141 females and 109 males, average age 59 (32–96); the procedure breakdown is shown in Table 1 along with the average tourniquet time for the procedure and any complications. A total of 3 postoperative complications were identified, only 1 of which was directly related to the tourniquet's use (1 neuropraxia which resolved by 6 months, 2 superficial wound infections), and the use of the tourniquet was discontinued intra-operatively in 1 case as a result of a venous tourniquet effect and inadequate bloodless field.

## 4. Discussion

In our experience, the S-MART tourniquet provides effective exsanguination and bleeding control when used in minor upper limb surgery. We experienced very few complications as a result of its use as suggested in Noordin's article, and we feel that its benefits certainly outweigh any risks in its use.

In particular, we feel that this type of tourniquet provides the following advantages.

*Decreased Tourniquet Times*. Whilst we have no data to confirm our thoughts, anecdotally we certainly feel that application of the tourniquet after formal draping of the patient reduces wasted time with the pneumatic tourniquet inflated after nonsterile exsanguination, and the application technique is faster than that using an esmarch bandage for sterile exsanguination.

*Less Interference with the Operative Field*. Whilst not a particular problem with hand and wrist surgery, for procedures involving more proximal extension of the operative field the ring type sterile tourniquet provides benefit in terms of its nonmigratory character. Large pneumatic tourniquets have a tendency to slip distally on the limb and encroach on the surgical field; we found this not to be a problem with the S-MART.

*Reliable Tourniquet Effect.* Effective use of a pneumatic tourniquet relies upon adequate exsanguination, proper tourniquet placement, and a good pressure seal between the tourniquet and pressurisation machine. In our experience, failure in one of these three aspects often leads to an inadequate bloodless field. The constricting elastic ring in the S-MART is factory made, and the occlusion pressure is preset. There is a choice of tourniquet depending on the desired limb occlusion pressure and the size of the limb to which it will be applied. This gives a more reliable occlusive effect on the arterial supply to the limb whilst not exerting a pressure that is so high as to cause local soft tissue problems.

Downsides to the use of this type of tourniquet are that the pressure cannot be released temporarily during the procedure as this requires division of the silicone ring with a scalpel; also it cannot be used in cases where there is an unstable fracture in the affected limb due to the risk of embolisation.

## 5. Conclusion

In our experience, the use of the S-MART exsanguination tourniquet is an effective and safe way to provide a bloodless field in hand and wrist surgery. Its use in our department in 250 cases did not indicate a higher tourniquet-related complication rate when compared to the use of a pneumatic tourniquet as suggested by Noordin et al. The use of S-MART is being driven by the infection control benefits of single-patient use coupled with the creation of a superior bloodless field, improved access to operative sites, versatility of site placement, and time saving capabilities.

## Figures and Tables

**Table 1:** Table of results.

Procedure	Number	Tourniquet time	Inadequacy	Complication
Carpal tunnel decompression	159 unilateral, 37 bilateral	6 mins	1 venous tourniquet	1 wound infection
Trigger finger release	27	7 mins		1 wound infection
Ganglion excision	8	9 mins		
De Quervain's decompression	5	11 mins		
